# The Swiss Prison Study (SWIPS): Protocol for Establishing a Public Health Registry of Prisoners in Switzerland

**DOI:** 10.2196/23973

**Published:** 2020-12-08

**Authors:** Thomas Gaisl, Naser Musli, Patrick Baumgartner, Marc Meier, Silvana K Rampini, Eva Blozik, Edouard Battegay, Malcolm Kohler, Shekhar Saxena

**Affiliations:** 1 Global Health and Population Harvard T H Chan School of Public Health Boston, MA United States; 2 Department of Internal Medicine University Hospital Zurich Zurich Switzerland; 3 Department of Pulmonology University Hospital Zurich Zurich Switzerland; 4 Department of Health Sciences Helsana Insurance Group Zürich Switzerland

**Keywords:** public health, prison medicine, epidemiology, health register

## Abstract

**Background:**

The health aspects, disease frequencies, and specific health interests of prisoners and refugees are poorly understood. Importantly, access to the health care system is limited for this vulnerable population. There has been no systematic investigation to understand the health issues of inmates in Switzerland. Furthermore, little is known on how recent migration flows in Europe may have affected the health conditions of inmates.

**Objective:**

The Swiss Prison Study (SWIPS) is a large-scale observational study with the aim of establishing a public health registry in northern-central Switzerland. The primary objective is to establish a central database to assess disease prevalence (ie, International Classification of Diseases-10 codes [German modification]) among prisoners. The secondary objectives include the following: (1) to compare the 2015 versus 2020 disease prevalence among inmates against a representative sample from the local resident population, (2) to assess longitudinal changes in disease prevalence from 2015 to 2020 by using cross-sectional medical records from all inmates at the Police Prison Zurich, Switzerland, and (3) to identify unrecognized health problems to prepare successful public health strategies.

**Methods:**

Demographic and health-related data such as age, sex, country of origin, duration of imprisonment, medication (including the drug name, brand, dosage, and release), and medical history (including the International Classification of Diseases-10 codes [German modification] for all diagnoses and external results that are part of the medical history in the prison) have been deposited in a central register over a span of 5 years (January 2015 to August 2020). The final cohort is expected to comprise approximately 50,000 to 60,000 prisoners from the Police Prison Zurich, Switzerland.

**Results:**

This study was approved on August 5, 2019 by the ethical committee of the Canton of Zurich with the registration code KEK-ZH No. 2019-01055 and funded in August 2020 by the “Walter and Gertrud Siegenthaler” foundation and the “Theodor and Ida Herzog-Egli” foundation. This study is registered with the International Standard Randomized Controlled Trial Number registry. Data collection started in August 2019 and results are expected to be published in 2021. Findings will be disseminated through scientific papers as well as presentations and public events.

**Conclusions:**

This study will construct a valuable database of information regarding the health of inmates and refugees in Swiss prisons and will act as groundwork for future interventions in this vulnerable population.

**Trial Registration:**

ISRCTN registry ISRCTN11714665; http://www.isrctn.com/ISRCTN11714665

**International Registered Report Identifier (IRRID):**

DERR1-10.2196/23973

## Introduction

### Background

In institutions such as prisons and correctional facilities, health risks are disproportionately widespread. The World Health Organization in the European Region has estimated that approximately 6 million people are incarcerated every year in this region [1]. The health profile of the people in prison is complex and the risk factors for poor health often overlap with the risk factors for incarceration [2]. Cooccurring physical and mental health conditions are often found in close association to entrenched and intergenerational social disadvantage [3]. Data indicate that, in general, prisoners are sicker when compared with counterparts of the same age, race, and sex in a free society [4]. Additionally, prisons and detention centers are known to reduce the resources for inmates substantially, and disease frequencies and the health of individual prisoners are poorly understood owing to the lack of original data [1]. Further, many inmates lack opportunities to articulate themselves (eg, language barriers in Europe), and access to the health care system is extremely limited. Prison physicians often speak of a “blind spot in society” [5]. In contrast to the investigations of the judicial branch, which is obliged to publish numbers on the origin, age, sex, and penalties of convicts, systematic investigations on the health issues of inmates are extremely rare.

### Current Situation

Only a few prevalence studies conducted in the United States and Europe indicate that the disease profile of prison inmates is significantly different from that of the general population. In line with what is generally known about the health of the people in prison, data from the Health in Prisons European Database paint a picture of an extremely vulnerable population that experiences poor health and engages in risky health behaviors, leading to noncommunicable and communicable diseases and mental health conditions [1,6]. In this setting, drugs and nonmedical use of conventional medications seem to play a crucial role in the health of the people in prison [6]. In 2014, a report from the European Monitoring Centre for Drugs and Drug Addiction (EMCDDA) suggested that besides health-related problems, illicit drug use is highly prevalent among people in prison [7]. More recently, the EMCDDA has warned about the rapidly developing phenomenon of the use of new psychoactive substances (NPS) among prisoners [8]. Monitoring data indicate that prisons are the epicenter of this phenomenon and there is growing concern that NPS may be responsible for a large share of drug-related problems in prison [8]. Approximately 4%-56% of all inmates use psychoactive substances, but the exact percentage requires further investigation [4,9]. For comparison, there is ample evidence that the “opioid crisis” in the United States affects inmates in particular [10]. Currently, only limited data from European institutions are available [8]. Studies conducted in the United States estimate that the “off-label” usage of drugs in prisons is about 36.2% [11]. However, no comparable data from Switzerland are available and there seems to be a lack of systematically collected and comparable data on the health of incarcerated people.

In 2017, approximately 71% of prison inmates in Switzerland were foreigners and most were imprisoned in the Canton of Zurich [12]. One way of systematically recording specific aspects of the disease burden in this population is to analyze medical files and histories. From studies performed in the early 2000s, it has been established that analysis of medical records in prisons is an extremely reliable source. In fact, in some areas of disease burden (eg, infectious disease, assessment of cardiovascular risk), this can be considered superior to information gained from questionnaires or personal interviews [9,13]. Most importantly, a systematic collection and analysis of medical records has not yet been conducted for inmates in Switzerland.

### Police Prison Zurich

**Figure 1:**
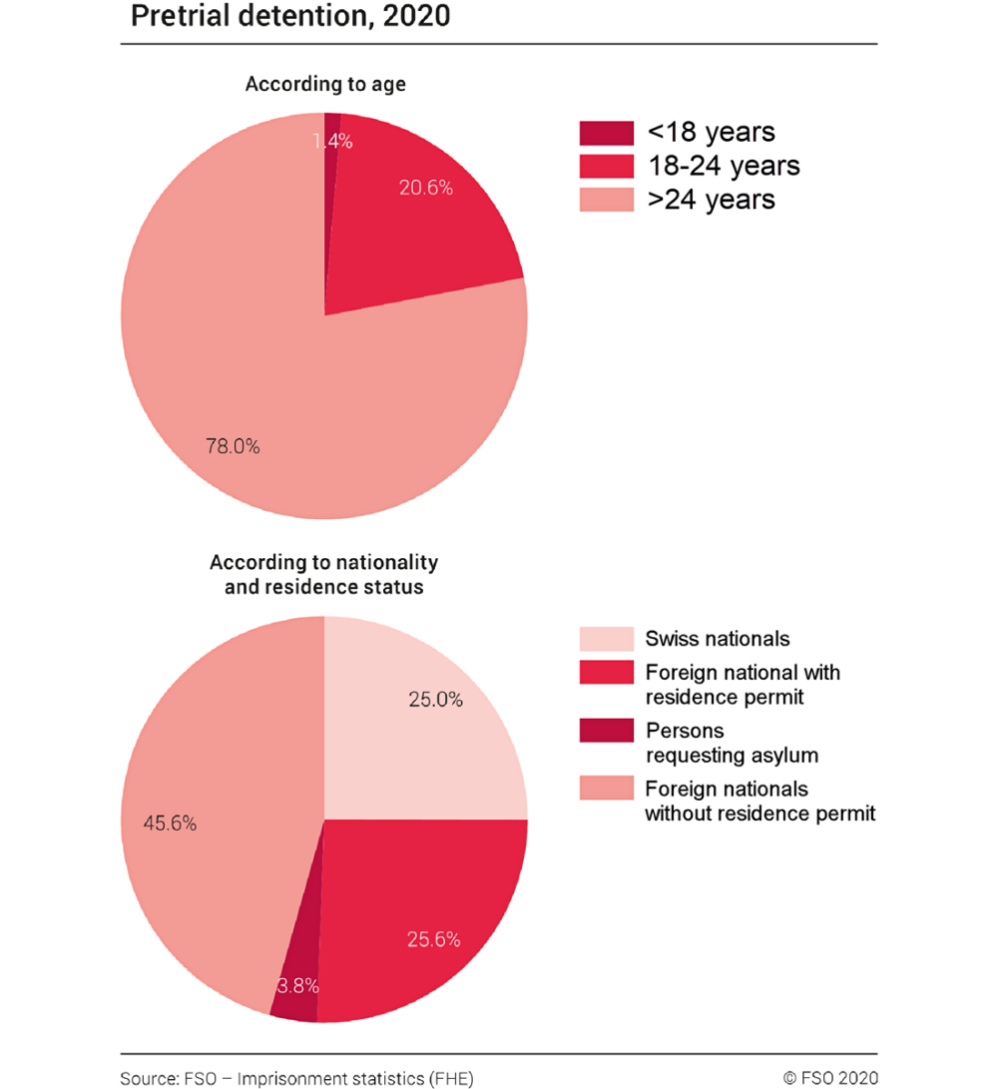
Imprisonment statistics for pretrial detention in Switzerland for year 2020 from the Federal Statistical Office.

Prison facilities are required to ensure adequate health care in both the somatic and psychiatric fields. The University Hospital Zurich has taken over the responsibility for providing somatic and psychiatric care to the Police Prison Zurich (PPZ) inmates. The PPZ is the largest inmate correctional facility in Switzerland, and approximately 10,000 to 12,000 prisoners are admitted per year. Data from the Federal Statistical Office in Switzerland indicate that inmates held for pretrial detention are predominantly young and male, and approximately 75% are non-Swiss nationals (Figure 1) [12]. Anecdotal evidence suggests that a large proportion of inmates (eg, refugees) have reported previously using drugs that were prescribed for off-label purposes. One example is the prevalent purchase of the substance *pregabalin* via web-based pharmacies [14]. Other examples include the underestimated usage of recreational drugs (eg, clonazepam) and other NPS [15]. Most of these drugs have potentially substantive health consequences, mainly attributed to their high potential for addiction and adverse effects. Furthermore, little information has been established on how recent refugee patterns may have affected the health conditions of the inmates in recent years.

### Hypothesis

In summary, the prison population is very vulnerable; yet, hardly any reliable data are available on the health of inmates [1,3]. The current geopolitical situation appears to exacerbate the problem as patients with a disproportionately poor health status have contact with the Swiss health care system. Data from the PPZ offer a unique opportunity globally for a representative analysis of the disease profile of inmates. The aim of this study is to collect health-related data in order to give insight into an unexplored area and underserved patient population. First, we hypothesize that the disease profile of inmates substantially differs from that of the general population. Specifically, certain chronic and psychiatric diseases are disproportionately prevalent among inmates and refugees. Second, a large number of drugs (eg, NPS) may be used “off-label,” and the origin of the inmates and the refugee route may have an impact on the disease profile of the affected. Third, recent geopolitical changes have significantly affected the overall disease profile of inmates.

## Methods

### Study Design

This is an observational study with a population-based cross-sectional baseline measurement and no prospective follow-up.

### Study Objectives

The primary objective is to establish a central database to assess the prevalence of diseases (ie, International Classification of Diseases [ICD]-10 codes [German modification]) among prisoners. The secondary objectives are (1) to compare the 2015 versus 2020 disease prevalence among inmates against a representative sample from the local resident population, (2) to assess longitudinal changes in disease prevalence from 2015 to 2020 by using cross-sectional medical records from all inmates at the PPZ, and (3) to identify unrecognized health problems to formulate models for successful public health strategies. Achieving these objectives can result in new insights into public health and in the construction of a valuable database of information regarding the health of inmates and refugees in Swiss prisons, thereby enabling future interventions in this vulnerable population.

### Study Questions

The main questions under investigation are as follows.

1. What is the health status of the inmate population and which conditions are overrepresented and underrepresented in the prison setting? In particular, how do psychiatric diseases affect inmates and what is the estimated prevalence of substance abuse in the prison population? Which infectious diseases (eg, tuberculosis, pneumonia, scabies) are highly prevalent among inmates and what are the comorbidities of this population?
2. What are the dynamics in the health profile of inmates (and thus refugees) during the European migrant crisis from 2015 onwards and how does the epidemiology of the prison population affect the balance between acute and chronic conditions?
3. Are there underrecognized health problems among vulnerable populations (eg, children, pregnant mothers, patients with suicidal ideation) or overrepresented populations (eg, body-packers, patients with traumatic injuries).

### Study Site

The health registry is based at the PPZ (Kasernenstrasse 29, 8021 Zurich, Switzerland). In this prison, all inmates of the Canton of Zurich are detained for up to a maximum of 7 days. Thereafter, prisoners are transferred to any of the following systems: (1) pretrial detention, (2) serving a particular prison sentence in a long-term facility, (3) anticipatory execution of sentence, or (4) subjection to coercive measures based on Aliens Act. The PPZ is the only central police prison in the Canton of Zurich serving a population of 1,520,968 (as of December 31, 2018), which is the most populous canton in the country. This prison has approximately 10,000 to 12,000 inmates yearly, of which about a third require medical attention (based on historical data from the PPZ/not published). The entire spectrum of medical attention needed to meet inmates’ medical needs is covered within the PPZ, such as general internal medicine (eg, asthma), traumatic injuries, psychiatric emergencies, and infectious diseases (eg, HIV).

### Participants

This study consists of prisoners within the PPZ from April 1, 2015 until August 31, 2020. The final sample will be exhaustive. All inmates within this timeframe will be included in this study and there will be no random selection. Only in documented cases wherein a prisoner allegedly refuses to participate in health-related activities, he or she will be excluded from the study and his or her data will not be analyzed. Otherwise, there are no exclusion criteria. Extrapolating from current numbers (approximately 850 prisoners per month × 64 months), it is expected that the final database will contain data from approximately 50,000 to 60,000 prisoners.

### Cooperation

The study will be conducted in cooperation with the Cantonal Police Zurich (contact person, Ms. Iris Suter), the University Hospital Zurich, which is the designated medical authority for the PPZ, and the Helsana AG (contact person, Dr. Eva Blozik). The Helsana AG is a major Swiss health insurance company with 2.1 million customers insured and will provide data on a matched Swiss cohort. Data will be extracted from the *Helsana Drug Report* [16], which is created in cooperation with the University Hospital Basel and the Institute for Pharmaceutical Medicine at the University of Basel. Another study site includes the University Hospital Zurich, Switzerland, which contractually provides all outpatient and inpatient medical care for the PPZ, including psychiatric consultations. No other parties are involved in the medical care of the PPZ prisoners.

### Data Collection and Management

Data entry into the health registry will be performed synchronously or asynchronously by a study team of physicians (TG, NM, PB, and MM) after carefully reviewing the source documents. A unique electronic case report form based on the variables in Table 1 and Table 2 was developed at the inception of this study. All source documents must be placed within the medical history of the prisoner. Table 1 and Table 2 summarize both the parameters and domains used in the study, which includes all variables, and other data extracted from the health registry. Unclear documentation will be discussed among the study team and incomplete data will be made apparent in the registry. After data entry, data will be checked for completeness and plausibility by the data manager. The database will be encrypted and only study personnel will have access to the source documents, which will not leave the prison. Encrypted backups of the database will be periodically conducted using external hard drives.

**Table 1:** Summary table of the general domains and parameters, which will be part of the registry.

Domain, subcategories		Parameter	Comment
**General characteristics**			
	Age	Years	At baseline
	Sex	Male/Female	Self-reported
	Date of imprisonment	Date	Data obtained from police
	Length of imprisonment	Days (n)	Data obtained from police
	Country of origin	Sovereign state according to the United Nations	Data obtained from police
**Diagnosis**			
	Principal diagnosis	ICD^a^-10 code	German modification of the ICD
	Secondary diagnosis (up to 20)	ICD-10 code	German modification of the ICD
**Medication**			
	Medication according to external documents	Active agent, dosage, administration	With external verification of drugs with dispensing category “A+”^b^
	Daily medication	Active agent, dosage, administration	With external verification of drugs with dispensing category “A+”
	On-demand medication	Active agent, dosage, administration	With external verification of drugs with dispensing category “A+”

^a^ICD: International Classification of Diseases.

^b^Category A+ according to the Federal Act on Medicinal Products and Medical Devices (Therapeutic Products Act) Article 24 (eg, opioids, benzodiazepines, methylphenidate).

**Table 2:** Summary of the systematic characteristics that will be assessed in the registry.

Characteristics, tests		Response	Explanation
**Particular characteristics at admission**			
	Reason for consulting a physician during the imprisonment	Text	Self-reported
	Evidence of a traumatic injury at admission	Yes/No	If applicable, then nature of the injury
	Need for a “Hafterstehungsfähigkeit”^a^	Yes/No	Data obtained from police
	History of illicit drug abuse	Yes/No	Data obtained from police
	Participant in the opioid replacement therapy program	Yes/No	Data obtained from police
	History of benzodiazepine dependence	Yes/No	Data obtained from police
	History of alcohol dependence	Yes/No	According to the DSM-IV^b^ criteria
	Result of the alcohol breath test at admission	mg/L	Optional
	Evidence of body packing (according to computed tomography scan results)	Yes/No	Data obtained from hospital
**Particular characteristics during the imprisonment**			
	Need to consult a psychiatrist during imprisonment	Yes/No	Data obtained from police
	Admission to a hospital during imprisonment	Yes/No	Data obtained from police
**Special reports**			
	Blood pressure chart	Source document	Optional
	Blood sugar chart	Source document	Optional
**Medical history**			
	External documents	Source document	Optional
	Laboratory results (including pregnancy test)	Source document	Optional

^a^Hafterstehungsfähigkeit: initial 24/7 medical assessment by a physician, after which it is decided whether the convict can be admitted to the prison in the first place.

^b^DSM-IV: Diagnostic and Statistical Manual of Mental Disorders, Fourth Edition.

### Quality Control

For this health registry, the following 4 quality control measurements will be implemented, and the results will be provided in the final manuscript:

1. Interobserver variability: The source documentation of 150 random patients will be processed by 2 physicians (ie, 450 degrees of freedom for every variable will be assessed) and disparities observed with regard to all domains will be summarized.
2. Matching with a local database from the jurisdiction: Data from the health registry will be compared with data from the jurisdiction, and discrepancies (eg, country of origin) will be further investigated by the study team.
3. Internal validity: The consumption of medications will be summarized for each month from 2015 to 2020, and these data will be compared with the prescription data collected from the PPZ’s local pharmacy, which collects monthly data for all dispensed medications. This step ensures that the actually dispensed medication is equal to the prescribed medication.
4. Verification of the source documents: Foreign health-related documents must be checked and, if necessary, translated to German by a recognized body (eg, translation services). In case of an already established course of pharmaceutical drug treatment with the dispensing category “A+,” according to the Federal Act on Medicinal Products and Medical Devices (Therapeutic Products Act) Article 24 (eg, opioids, benzodiazepines, methylphenidate), they require an external verification of the original prescription and a statement from the physician. Written verifications by the federal authorities or nongovernmental organizations involved in the opioid replacement therapy program will also be accepted. Other verifications by nongovernmental organizations (eg, refugee organizations) will not be accepted.

### Data Protection

In this registry, anonymized health data will be analyzed and stored. Other sensitive data, particularly, religious, political, social security, or administrative data will not be included as part of this study. The anonymized data sets generated or analyzed during this study will be available upon request from TG. Data are handled according to the Schengen Data Protection Act.

### Statistical Considerations

Statistical analyses will be conducted by the study team in consultation with senior statisticians. For baseline analyses, means or medians will be reported according to the distribution of the continuous variable. Proportions will be reported for categorical variables. For hypothesis testing, a two-sided *t* test for normally distributed continuous variables and nonparametric tests for nonnormally distributed continuous variables will be conducted; chi-square testing will be used for categorical variables. Linear or logistic regression models with or without adjustment for potential confounders (ie, age, sex, nationality) will be used when appropriate. Regression analysis estimates will be reported using 95% confidence intervals, and a two-sided *P* value of <.05 will be considered statistically significant for all reported tests. For the longitudinal data, a generalized linear mixed model with random effects (accounting for the nationality of the prisoner during the refugee crisis) will be used when appropriate. Statistical analyses will be performed using STATA Version 16 (StataCorp LP).

### Outcomes

The primary outcome measure will be disease prevalence by using cross-sectional data from prisoner’s medical records (ie, using the ICD-10 codes [German modification]) from 2015 to 2020. Secondary outcome measures include the following: (1) A comparison of disease prevalence (ICD-10 codes [German modification]) of the inmates at the PPZ to that of a representative sample from the local resident population by using cross-sectional medical records from all inmates at the PPZ from 2015 to 2020 and (2) longitudinal changes in disease prevalence (ICD-10 codes [German modification]) from 2015 to 2020 by using cross-sectional medical records from all inmates at the PPZ.

### Ethical Aspects

This study was approved by the ethical committee of the Canton of Zurich. The committee will be informed about major changes to the protocol in agreement with local requirements. This study will be conducted in accordance with the protocol and the principles enunciated in the current version of the Declaration of Helsinki, the guidelines of Good Clinical Practice issued by the International Conference on Harmonization, and the requirements from the Swiss Law and Swiss regulatory authorities. This study protocol is reported according to the SPIRIT (Standard Protocol Items: Recommendations for Interventional Trials) guidelines and results will be reported according to the STROBE (Strengthening the Reporting of Observational Studies in Epidemiology) statement from the University of Basel, Switzerland.

## Results

This study was approved on August 5, 2019 by the ethical committee of the Canton of Zurich with the registration code KEK-ZH No. 2019-01055 and funded in August 2020 by the “Walter and Gertrud Siegenthaler” foundation and the “Theodor and Ida Herzog-Egli” foundation. This study is registered with the International Standard Randomized Controlled Trial Number registry. Data collection started in August 2019 and results are expected to be published in 2021. Findings will be disseminated through scientific papers as well as presentations and public events.

## Discussion

This health registry is designed to provide new public health insights into a group of vulnerable patients. The implementation of the study protocol promises to allow for the identification of specific health problems to help prepare successful public health strategies to meet these challenges. To gain a representative sample from the Canton of Zurich inmate population, this study required a high enrollment. Inmates at the PPZ usually spend a minimum of 1 day to a maximum of 7 days in the facility; they are internationally mobile, often unable to speak German, and have no contact details provided. Gaining informed consent from each prisoner is a disproportionately large expenditure. In this observational study, we assume that the expected increase in knowledge will benefit future inmates and thus, outweigh the disadvantages associated with privacy concerns. Moreover, given these operational challenges, any large-scale study on the prisoner population will likely need to be conducted without receiving individualized informed written consent. Thus, in accordance with the Swiss Human Research Act Article 34, the need to obtain informed consent is waived for this study, and data collected from all prisoners will be gathered. Only in case of documented alleged refusal to participate in health-related activities, data from the prisoner will not be included in this study.

Currently, no similar observational studies on Swiss inmates are available. Data from the United States and European registries indicate that prisoners are sicker when compared with their counterparts in a free society [2-4]. However, these data are limited to a certain location and time and they do not investigate changes over time within a certain cohort. Preliminary studies suggest that illicit drugs and especially NPS are a growing problem in European prisons, but only limited data from European prisons are available [7,8]. This study will be able to draw conclusions on these issues based on a 5-year representative sample of approximately 50,000 to 60,000 prisoners from the PPZ. Thus, this study will add valid evidence to the current health status of prisoners, and findings can be used to promote healthy behaviors (eg, future intervention studies) or policy change for this population.

The findings of this study will be disseminated through peer-reviewed journals as well as national and international conference presentations. Collaborations with other researchers in the field will be promoted. The data that support the findings of this study are available from the corresponding author upon reasonable request. Furthermore, data will be presented to Swiss policymakers and health care workers to improve the public health of prisoners in Switzerland. In conclusion, this study will construct a valuable database of information regarding the health of inmates and refugees and will act as groundwork for future interventions in this vulnerable population.

## Abbreviations

EMCDDA: European Monitoring Centre for Drugs and Drug Addiction
ICD: International Classification of Diseases
NPS: new psychoactive substances
PPZ: Police Prison Zurich
